# Analysis of Gut Microbiota as a Diagnostic Biomarker for Lung Adenocarcinoma with Qi-Deficiency and Phlegm-Turbid Stagnation

**DOI:** 10.2174/0113862073303081240521083505

**Published:** 2024-06-05

**Authors:** Jiabin Chen, Qinqin Hu, Kequn Chai, Sheng Wang

**Affiliations:** 1 Department of Oncology, Tongde Hospital of Zhejiang Affiliated to Zhejiang University of Traditional Chinese Medicine, Hangzhou, Zhejiang 310012, China;; 2 The Second Clinical Medical College, Zhejiang Chinese Medicine University, Hangzhou, Zhejiang 310012, China;; 3 Respiratory Department, Jinhua Guangfu Cancer Hospital, Zhejiang 310053, China

**Keywords:** Lung adenocarcinoma, traditional chinese medicine, qi-deficiency and phlegm-turbid stagnation, gut microbiota, diagnostic model

## Abstract

**Background:**

In Lung Adenocarcinoma (LUAD), Qi-deficiency and Phlegm-turbid stagnation (QP) are the most prevalent Traditional Chinese Medicine (TCM) syndrome.

**Methods:**

Herein, we collected 90 fecal samples (Healthy individual (H): 30; other syndrome (O): 30; QP: 30) and explored the composition and diversity of gut microbiota in LUAD patients with QP syndrome using 16s-rRNA sequencing. Then, we identified biomarkers for QP syndrome in LUAD patients with Linear Discriminant Analysis (LDA) effect size (LEfSe) and applied logistic regression analysis to construct a diagnostic model evaluated with the area under the receiver operating characteristic curve (AUC) and validated with data from metagenomics.

**Results:**

The α diversity and β diversity revealed that the microbiota community structure in LUAD patients with QP syndrome was different from that with healthy individuals and LUAD patients with other syndromes. At the phylum level, the QP group had more abundance of Bacteroidetes and less Proteobacteria than the O group. At the genus level, the abundance of 4 genera (Bacteroides, Parabacteroides, Prevotella, and Flavonifractor) was different between the QP group and O group. Moreover, LEfSe indicated that those 4 genera might be the biomarkers for LUAD patients with QP syndrome. Then, we used those 4 genera to develop a diagnostic model. The AUC based on 16s-rRNA sequencing and metagenomics was 0.989 and 1, respectively.

**Conclusion:**

A diagnostic model was developed, which would be an available tool for the clinical diagnosis of LUAD with QP syndrome.

## 1 INTRODUCTION

The most frequent malignant tumor and the leading cause of cancer-related mortality worldwide is lung cancer (LC) [1, 2]. Non-small cell lung cancer (NSCLC) is the main subtype of LC (80-85%) and includes two major histological subtypes: squamous-cell carcinoma and adenocarcinoma [1-3]. Lung adenocarcinoma (LUAD) is the most heterogeneous and aggressive LC and accounts for over 40% of all LC cases [3]. While the development of diagnostic techniques and therapies such as immunotherapy have increased the overall survival time of LUAD patients, the 5-year survival rate is still less than 20% [4, 5]. In addition, considerable side effects often occur, such as immune pneumonia and gastrointestinal reactions, when patients are on medication [6]. Traditional Chinese medicine (TCM), as a complementary and alternative medicine therapy, has been widely applied for the treatment of LC in China and has shown good efficacy in improving the quality of life, reducing adverse effects, and prolonging survival time [7, 8].

There are more than 100 trillion microbes in the human gut, which are over 100 times more genes than the human genome [9, 10]. Changes in the composition and distribution of gut microorganisms contribute to several diseases, such as diabetes and cancers [11]. The specific role of gut microorganisms in the progression of cancers has been proven to be complex [12]. However, there is no doubt that gut microbes could contribute to the tumorigenesis and growth of cancers, which may have been caused by the regulating of endocrine, metabolic, inflammation, and immune systems [11, 12], and researchers have shown that gut microbes were highly associated with several cancers, including gastric, colorectal, pancreatic, hepatocellular, breast and prostate cancer [13]. Additionally, gut microorganisms could influence the efficacy of immune checkpoint inhibitors in LC [14, 15].

Syndrome differentiation is the key theory of TCM, meaning that corresponding therapies should be adopted for different TCM syndromes [16,17]. Many studies have reported that TCM could regulate the composition and distribution of gut microbes and mucosal barrier function to repress the development and progression of diseases, which may be a mechanism of TCM in treating diseases [18]. Qi-deficiency and Phlegm-turbid stagnation (QP) is one of the most common TCM syndromes in LC (15.8-21.9%) [19]. Previous studies have reported that in LUAD patients with Qi-deficiency and Phlegm-turbid stagnation syndrome, chemotherapy combined with therapeutic methods of eliminating Phlegm and supplementing Qi have better efficacy in clinical remission rate and alleviating the side effects of chemotherapy [19, 20]. Meanwhile, basic experiments showed that Chinese herbal medicine for eliminating phlegm and supplementing Qi could control the development of LC [21]. However, there has been no research indicating the composition and distribution of gut microbiota in LUAD patients with QP syndrome.

Previous research showed that gut microbes could be biomarkers for the diagnosis of many diseases, like esophageal squamous cell carcinoma, showing good diagnostic performance [22, 23]. 16s-rRNA is a highly conserved gene fragment in bacteria and archaea, with a certain degree of variability. By sequencing the 16S rRNA gene, different genera and species of bacteria can be identified in the microbial community, and their relative abundance can be evaluated with an economically efficient technique [23]. In the present study, we collected fecal samples from LUAD patients with QP syndrome and explored the composition and function of gut microbiota with 16s-rRNA. Then, we identified QP syndrome-specific biomarkers with the machine learning method, which may be conducive to the diagnosis of QP syndrome.

## 2 METHODS

A detailed experimental method is shown in Supplementary File **1**.

### 2.1 Subjects

All LUAD patients (QP syndrome: 40; Other syndromes (O): 40) and healthy individuals (H: 30) were collected from Tongde Hospital. All patients were diagnosed as LUAD according to the TNM staging system (8th edition). Three Professors of TCM judged the TCM syndrome based on clinical symptoms according to《Chinese Medical Constitution 2008 Issued by the Chinese Association of TCM》 and 《TCM Clinical Diagnosis and Treatment Terminology》. The study was approved by the ethics committee of Tongde Hospital (No. 2016-057).

### 2.2 Inclusion Criteria

1) Patients aged 18-75 years with histologically or cytologically confirmed LUAD, 2) Life expectancy >6 months, 3) Patients without surgical operation, chemotherapy, radiotherapy, biological therapy, or TCM treatment previously, 4) Patients with Karnofsky (KPS) ≥ 60 and Eastern Cooperative Oncology Group (ECOG) ≤2.

### 2.3 Exclusion Criteria

1) Patients with any severe acute or chronic medical condition, 2) Pregnant or lactating women. 3) Hemoglobin < 100g/L, white blood cell < 4*109/L, neutrophils < 2*109/L, platelet < 100g/L; Serum creatinine >1.5 ULN (upper limit of normal value); Hemobilirubin > 1.5 ULN, ALT or AST > 2.5 ULN. 4) Antibiotic therapy in the past 3 months.

### 2.4 Sample Collection

After collecting stool samples, DNA was extracted with the E.Z.N.A. ^®^Stool DNA Kit (D4015, Omega, Inc., USA) according to the manufacturer's instructions, then eluted in 50 μL of Elution buffer and stored at -80 °C.

### 2.5 16s-RNA Sequencing and Bioinformatics Analysis

16s-RNA sequencing and quality control of raw data were performed as described previously [24]. The V3-V4 regions of the 16S rRNA were amplified with primers (341F:5'-CCTACGGGNGGCWGCAG-3'; 805R: 5'-GACTACHVG GGTATCTAATCC-3'). 25 ng of template DNA, 12.5 μL PCR Premix, 2.5 μL of each primer, and PCR-grade water were mixed into a 25 μL reaction mixture, which was amplified with PCR. The conditions of PCR were described previously [24]. The products of PCR were purified by AMPure XT beads (Beckman Coulter Genomics, Danvers, MA, USA) and quantified by Qubit (Invitrogen, USA). The size and quantity of the amplicon pools, which were used for sequencing, were estimated with Agilent 2100 Bioanalyzer (Agilent, USA) and the Library Quantification Kit for Illumina (Kapa Biosciences, Woburn, MA, USA). The libraries were sequenced on the NovaSeq PE250 platform.

Samples sequencing was performed on an Illumina NovaSeq platform. Paired-end reads were linked to samples according to the unique barcode and then truncated and merged with FLASH. Low-quality raw reads and chimeric sequences were filtered under specific filtering conditions using the fqtrim (v0.94) and Vsearch software (v2.3.4), respectively. The feature was defined at a 99% similarity level. Blast performed the sequence alignment of species annotation, and the alignment database was SILVA and NT-16S. The functional prediction was performed with PICRUSt (v1.1.2) based on the Kyoto Encyclopedia of Genes and Genomes (KEGG) database.

### 2.6 Metagenome Analysis

As described previously [25], removing low-quality paired-end reads (Score <20 and length <50 bases) and mapping and aligning the paired-end reads to the human genome with IGC bowtie2 (v 2.3.0). Then, the number of mapped paired-end reads is counted and mapped to metaphlan2 to obtain and calculate the relative abundance of phyla, genera, and species profiles.

### 2.7 Statistical Analysis

Statistical analyses were performed with R 4.1.3 (https://www.r-project.org). The differences in quantitative data between the two groups were compared with the Wilcoxon rank-sum test. The α diversity was assessed with the Simpson and Shannon index. The β diversity was assessed by Principal coordinate analysis (PCoA) based on Bray-Curtis and Jaccard matrixes. Linear discriminant analysis (LDA) effect size (LEfSe) was applied to identify The taxa biomarkers were identified with Linear discriminant analysis (LDA) effect size (LEfSe) in the galaxy online analysis platform (http://huttenhower.sph.harvard.edu/galaxy/). Wilcoxon rank sum test: P<0.01 and the log value >3.0 were set as the cut-off values. The diagnostic model was built with logistic regression analysis and evaluated in the area under the receiver operating characteristic curve (AUC). The relationship between the two indexes was calculated with Person analysis.

## 3 RESULTS

### 3.1 Clinical Characteristics

110 participants (H: 30; LUAD: 80) were enrolled in the study. 80 LUAD patients included two groups: QP: 40; O: 40. 90 samples were subjected to 16s-RNA analysis: H: 30; QP: 30; O: 30. Twenty samples were analyzed with metagenomics (QP: 10; O: 10) to validate the results of 16s-RNA analysis. The basic information of all participants was summarized in Supplementary Table **1**.

### 3.2 Evaluation of Gut Microbiome based on 16s-RNA Analysis

#### 3.2.1 α Diversity and β Diversity

In total, 6 081 881 raw reads were obtained from 90 samples (H: 30; QP: 30; O: 30), and 9817 Features were matched, with an average of 57973±106 Features/sample. The value of the good's coverage estimator was 99.79%

**Fig. (1):**
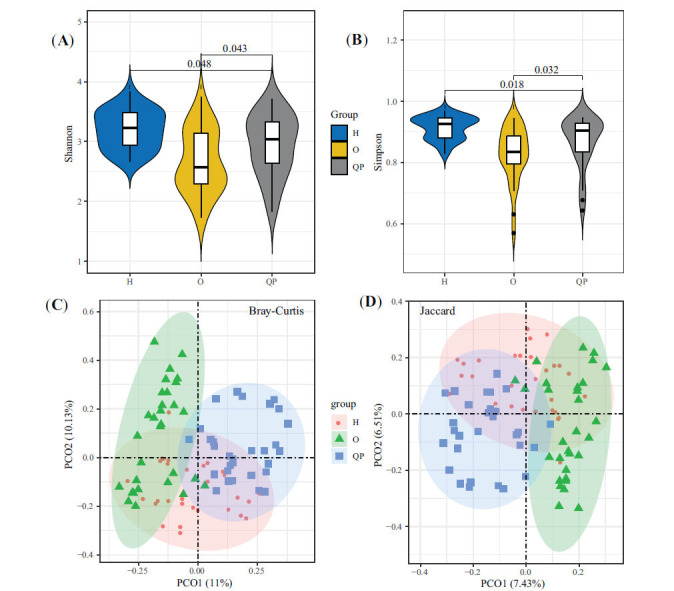
The assessment of Microbiota diversity and composition. (**A**) α diversity of gut microbiota measured by Shannon index, (**B**) by Simpson index. (**C**) β diversity of PCoA based on Bray-Curtis matrixes, (**D**) based on Jaccard matrixes. H: healthy individuals; QP: LUAD with Qi-deficiency and Phlegm-turbid stagnation; O: LUAD with other syndrome; PCoA: Principal coordinate analysis.

Then, we calculated the Shannon and Simpson index to assess the α diversity, which was used for the evaluation of microbiota community structure. As shown in Fig. (**1A**), the Shannon index of the gut microbiota of QP patients was significantly lower than that of healthy individuals (P=0.048) and higher than that of patients with other syndrome (P=0.043). Similar results were observed in the Simpson index (QP vs. H: P=0.018; QP vs. O: P=0.032, Fig. **1B**). The quality manager samples were found to be clustered in a comparatively independent zone by PCoA, albeit with some modest overlap with the other two groups (Fig. **1C** and **D**).

#### 3.2.2 Taxonomy Comparison of Gut Microbiota at the Phylum Level

**Fig. (2):**
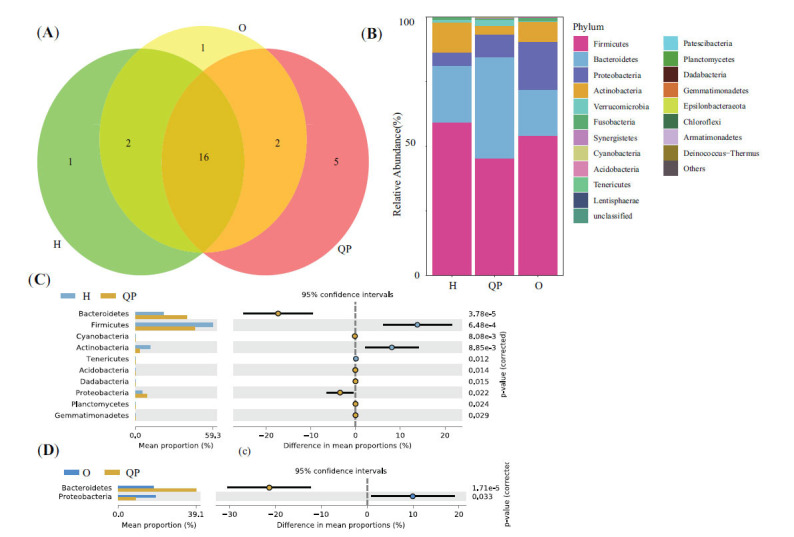
Taxonomy comparison of gut microbiota at phylum level. (**A**) shared 16 phyla exciting in the three groups. (**B**) Phylum distribution of gut microbiota in three groups. (**C**) Differences in gut microbiota between H group and QP group, (**D**) between O group and QP group.

At the phylum level, 16 phyla were found in the three groups simultaneously (Fig. **2A**). In addition, in all three groups, the most abundant microbiota was Firmicutes, followed by Bacteroidetes and Proteobacteria (Fig. **2B**). Compared with the H group, the abundance of Bacteroidetes, Cyanobacteria, Acidobacteria, Dadabacteria, Proteobacteria, Planctomycetes, and Gemmatimonadetes was increased in the QP group, whereas, the abundance of Firmicutes, Actinobacteria, and Tenericutes was opposite (Fig. **2C**). Compared with the O group, the QP group had more proportion of Bacteroidetes and less Proteobacteria (Fig. **2D**).

#### 3.2.3 Taxonomy Comparison of Gut Microbiota at the Genus Level

**Fig. (3):**
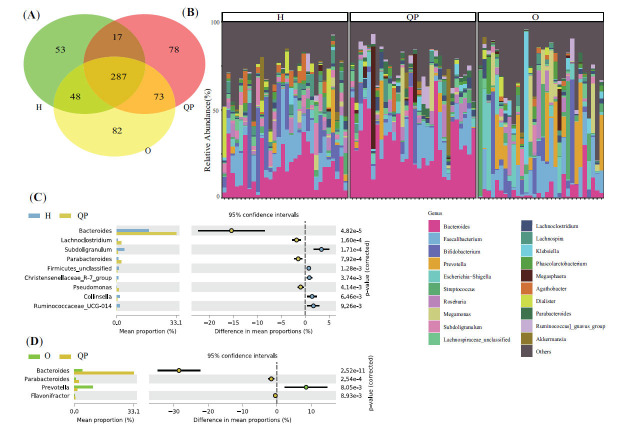
Taxonomy comparison of gut microbiota at genus level. (**A**) shared 287 genera exciting in the three groups. (**B**) Genus distribution of gut microbiota of all subjects. (**C**) Differences in gut microbiota between H group and QP group, (**D**) between O group and QP group.

At the genus level, 287 genera existed in the three groups simultaneously (Fig. **3A**). The highest proportion of genus in the QP group was Bacteroides, followed by Faecalibacterium and Bifidobacterium (Fig. **3B**). Similar phenomena were observed in H group. However, in the O group, the most abundant genus was *Faecalibacterium*, followed by *Prevotella* and *Streptococcus*. Then, we compared the difference of genera between the two groups with P<0.01 as the threshold. We found that the abundance of Bacteroides, *Lachnoclostridium, Subdoligranulum, Parabacteroides*, Christensenellaceae_R-7_group, *Pseudomonas*, *Collinsella*, and Ruminococcaceae_UCG-014 was significantly different between QP group and H group (Fig. **3C**). And, between QP group and O group, a significant difference in 4 genera (Bacteroides, *Parabacteroides*, *Prevotella*, and *Flavonifractor*) was found (Fig. **3D**).

#### 3.2.4 The Diagnostic Model for LUAD with QP Syndrome

**Fig. (4):**
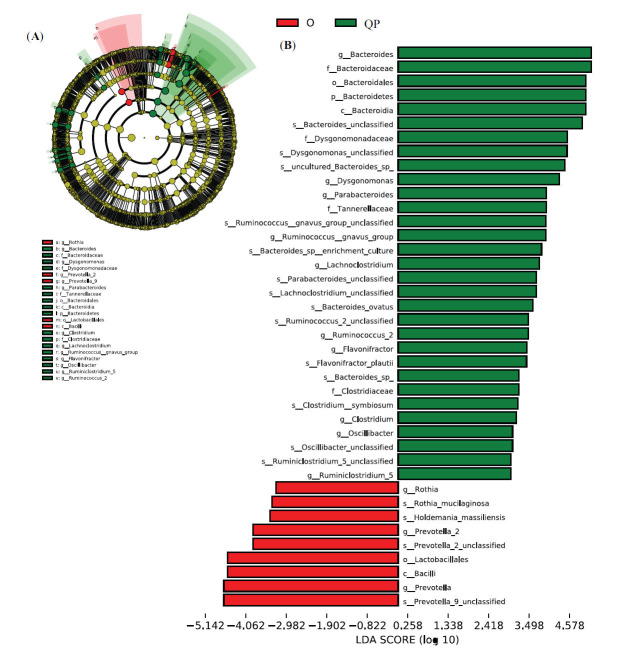
Identification of differently abundant taxonomy between O group and QP group using LEfSe analysis. (**A**) Cladogram showed the most differentially abundant taxa between the two groups. (**B**) The taxa meeting the cut-off value: P<0.01 and LDA>3. LEfSe: Linear discriminant analysis (LDA) effect size.

To investigate the variation of the microbial community composition between the QP and O group and determine QP syndrome-specific biomarkers, we performed LEfSe with P<0.01 and LDA>3 as the thresholds. At the genus level, 10 genera were increased in the QP group, and 3 genera were increased in the O group (Fig. **4**).

In addition, taxonomy comparison revealed that among 10 genera enriched in the QP group, 4 genera (*Bacteroides*, *Parabacteroides*, *Prevotella*, and *Flavonifractor*) were increased in the QP group, compared with that in the O group. Hence, we selected those 4 genera for further research. Next, we constructed a co-occurrence network of 4 genera with Pearson analysis. The cut-off values were P<0.01 and lcorl>0.4. The co-occurrence network of 4 genera is shown in Fig. (**5A**). The distribution of 4 genera in all samples is presented in Fig. (**5B**).

**Fig. (5):**
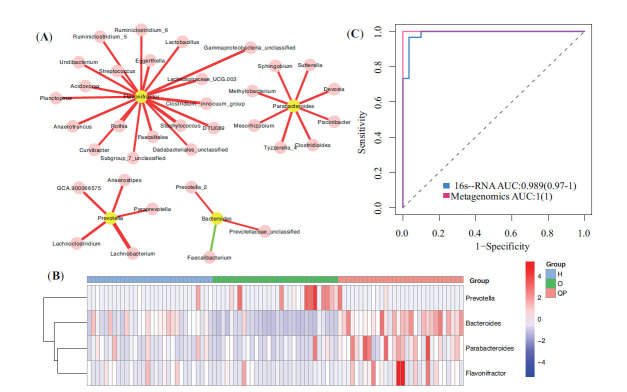
Development of a diagnostic model with 4 genera (Bacteroides, Parabacteroides, Prevotella, and Flavonifractor). (**A**) The co-occurrence network of 4 genera. The node represents genus. The edge represents connection, and red and green indicates positive and negative correlation. The thicker the edge, the closer the correlation. (**B**) The AUC of the diagnostic model. (**C**) The distribution of 4 genera in all samples. AUC: the receiver operating characteristic curve.

The LEfSe analysis and taxonomy comparison demonstrated that those 4 genera were enhanced in the QP group, hinting that those 4 genera may be diagnostic biomarkers of QP syndrome in LUAD. Hence, we applied logistics analysis to analyze those 4 genera and develop a diagnostic model for QP LUAD. The AUC of the diagnostic model was 0.989 (Fig. **5C**). The diagnostic formula was as follows.


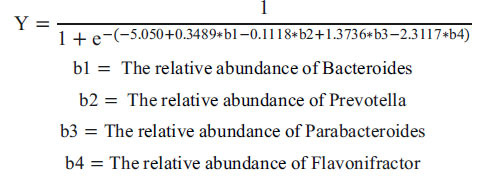


#### 3.2.5 Functional Annotations of Gut Microbiota

For predicting the biological function altered by different gut microbiota between QP and O groups, we performed PICRUSt analysis with FDR<0.01 as the cut-off value and found that between QP and O group, 103 pathways were enriched (Supplementary Fig. **1**). Then, we explored the relationship between 103 pathways and 4 genera (*Bacteroides*, *Parabacteroides*, *Prevotella*, and *Flavonifractor*) with Pearson analysis. Fig. (**6**) showed the pathways highly related to 4 genera (P<0.01; lcorl>0.4).

### 3.3 Validation of the Diagnostic Model

**Fig. (7):**
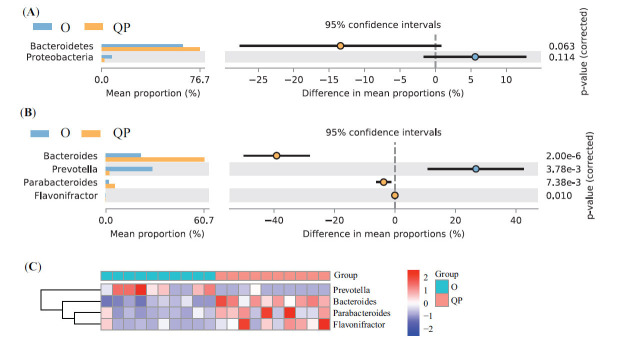
Validation of diagnostic model with Metagenomics. (**A**) Differences in gut microbiota between O group and QP group at phylum level, (**B**) at genus level. (**C**) The distribution of 4 genera in all samples.

Then, we applied Metagenomics to analyze 20 stool samples (QP: 10; O: 10). The Shannon and Simpson index were increased in the QP group (Supplementary Fig. **2A**). Additionally, the PCoA illustrated statistical difference between the O and QP group (Supplementary Fig. **2B**). At phylum Level, there was no statistical difference (Fig. **7A**). At genus level, consistent with previous finding, the proportion of Bacteroides, Parabacteroides, and Flavonifractor was increased in QP group, and Prevotella was decreased (Fig. **7B**). The distribution of 4 genera was presented in Fig. (**7C**). Then, we used those 4 genera to validate the diagnostic model. The AUC based on metagenome analysis was 1 (Fig. **5C**).

## 4 DISCUSSION

Recently, the gut microbiota has been proven to play a vital role in host health. Changes in the diversity and composition of gut microbiota could promote or release the progression of several diseases, including cancers, and could be a diagnostic marker for some illnesses [11, 12]. In China, TCM has been widely used for the prevention and therapy of diseases for many years and has shown good efficacy. In addition, many researches have shown that oral TCM could influence the structure of gut microorganisms, which in turn will affect the digestion and absorption of TCM, indicating that TCM can also treat diseases by regulating the gut microbiota of the host [16, 18]. According to the core theory of TCM Syndrome Differentiation Treatment, the same disease can be classified into different syndromes, which should be treated differently [16, 17]. However, it is difficult to discriminate TCM symptoms only by clinical symptoms, and lack of laboratory diagnosis and typical clinical biomarkers increases the difficulty. One of the most prevalent TCM syndromes in LC is QP symptoms [19]. The therapeutic approaches for removing phlegm and boosting Qi have shown better efficacy in the clinical remission rate and in mitigating the side effects of chemotherapy in LUAD patients with QP syndrome [19, 20]. For example, a real-world study demonstrated that the application of TCM for removing phlegm and boosting Qi had the potential antitumor progression effect and delayed the occurrence of acquired resistance [26]. A retrospective study found that Comprehensive CM treatments could significantly reduce progression risk, improve prognosis, and prolong survival time for patients with advanced LUAD with QP syndrome [27].

Herein, we selected the typical syndrome of LUAD patients, Qi-deficiency, and Phlegm-turbid stagnation (QP) as the research objects and collected and analyzed the fecal sample to reveal the diversity and composition of gut microbiota. Compared with healthy individuals (H), the Shannon and Simpson index of the QP group was decreased. However, they were higher than that of LUAD patients with other syndromes (O), hinting that the richness and homogeneity of gut microbiota in QP LUAD patients differed from healthy people and other syndrome LUAD patients. It was confirmed by PCoA. The top 3 abundant genera were *Bacteroides*, *Faecalibacterium*, and *Bifidobacterium*. Unlike the H group and O group, the abundance of *Genus-Bacteroides* and Parabacteroides was significantly elevated in the QP group. In addition, the QP group had a proportion of *Genus-Flavonifractor*. Previous studies have proven that *Bacteroides* was the most abundant bacteria at the genus level in healthy individuals, which was in line with the findings of this study [28, 29]. Compared with healthy people, lung cancer patients had a lower abundance of *Bacteroides*. Herein, we found that the abundance of *Bacteroides* in the QP group was higher than that in the H group, whereas in the O group, it was the opposite. *Genus-Bacteroides* is a double-edged sword that could stabilize intestinal mucosa, regulate carbohydrate metabolism, and activate the T-cell-dependent immune response [30]. However, it can also lead to evasion of host immune response endotoxin production, related to several diseases [31]. For example, *Bacteroides* produce Polysaccharide A against colorectal cancer *via* TLR2 signaling [32]. Nevertheless, it also could induce the stemness of colorectal cancer to promote colorectal carcinogenesis [31]. So far, no studies have reported the specific effects of *Bacteroides* on LUAD. *Genus-Parabacteroides* play a positive role in glucose and lipid metabolism and significantly correlate negatively with obesity, Nonalcoholic fatty liver disease, and diabetes mellitus [33]. In addition, a study reported that resistant starch increases *Genus-Parabacteroides* against colitis-associated colorectal cancer in rats [34]. *Genus-Prevotella* is present in humans, which is conducive to the breakdown of protein and carbohydrates and could be an opportunistic pathogens [35]. Modulation of levels of *Genus-Prevotella* therapeutically in the host either improves metabolism or reduces the risk of inflammation [35]. *Genus-Flavonifractor* could cleave the C-ring of the flavonoid molecules to degrade flavonoids, which play a vital role in the prevention of cancer, type 2 diabetes, cognitive dysfunction, and cardiovascular disease [36]. In turn, Flavonoids could influence the structure of the microbiota community by promoting beneficent bacteria and reducing pathogenic bacteria [36].

Then, we identified QP syndrome-specific biomarkers with LEfSe and found that all of those 4 genera (*Bacteroides*, *Parabacteroides*, *Prevotella*, and *Flavonifractor*) were with LAD>3 and P<0.01, meaning that those 4 genera could be QP syndrome-specific biomarkers in LUAD. In addition, the co-occurrence network of 4 genera showed 19 genera were highly related to *Flavonifractor*, 8 genera with *Parabacteroides*, 5 genera with *Prevotella*, and 3 genera with *Bacteroides*, hinting at the core position of these genera in LUAD with QP syndrome. Next, we applied those 4 genera to establish a diagnostic model with logistics analysis. The AUC was 0.989, which was similar to that based on Metagenomics (AUC=1), suggesting that this diagnostic model was suitable for diagnosing QP syndrome in LUAD patients and presented good diagnostic efficiency. This contributed to the diagnosis of TCM syndrome and guided the selection of clinical drugs, providing a theoretical basis for clinical medication. There are studies using machine learning methods to construct TCM syndrome diagnosis models for LC [37]. The model used subjective clinical symptoms as the main evaluation indicators, lacking objective experimental indicators, and the model included many indicators, making the operation relatively complex. In addition, studies used decision trees to screen out six plasma proteins to construct a diagnosis model for non-small cell lung cancer with qi deficiency, phlegm dampness, and blood stasis syndrome [38]. Similarly, this model lacks external data validation, and the AUC value of the model is only 0.79, which is significantly lower than the AUC value in this experiment. This study constructed a diagnostic model for LUAD with QP syndrome. The model was stable and reliable and can be used for subsequent syndrome diagnosis. This method also provides a reference for establishing the TCM syndrome diagnosis model.

Limitations: 1) Due to the diagnostic model being developed based on costly and time-consuming high throughput sequencing, it is difficult to popularize in clinical applications. 2) In the study, only 30 and 10 QP syndrome LUAD patients were used to construct and validate the diagnostic mode, which may lead to selection bias. In addition, we excluded some samples, like KPS ≥60. It may bring biased prognosis predictions. Therefore, it needs to be validated by large-scale clinical trials that include diverse samples. 3) No experimental research was conducted to testify to the role of gut microbiota, especially 4 genera (*Bacteroides*, *Parabacteroides*, *Prevotella*, and *Flavonifractor*). Hence, further investigation is required.

## CONCLUSION

The present study revealed the diversity and composition of gut microbiota in LUAD patients with Qi deficiency and Phlegm-turbid stagnation. Then, we identified 4 genera (*Bacteroides*, *Parabacteroides*, *Prevotella*, and *Flavonifractor*) as the biomarkers and developed a diagnostic model for QP syndrome in LUAD patients, which would be an available tool for clinical diagnosis of QP syndrome, and provide a new target for natural drug treatment of LUAD.

## Figures and Tables

**Fig. (6):**
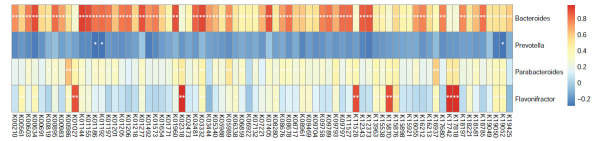
The pathways that were highly related to 4 genera (Bacteroides, Parabacteroides, Prevotella, and Flavonifractor) (P<0.01; lcorl>0.4).

## Data Availability

The authors confirm that all data generated or analyzed during this study are included in this published article.

## LIST OF ABBREVIATIONS

AUC: The Receiver Operating Characteristic Curve
H: Healthy Individuals
LC: Lung Cancer
LDA: Linear Discriminant Analysis
LEfSe: LDA Effect Size
LUAD: Lung Adenocarcinoma
NSCLC: Non-Small Cell Lung Cancer
O: Other Syndrome
PCoA: Principal Coordinate Analysis
QP: Qi-Deficiency and Phlegm-Turbid Stagnation
TCM: Traditional Chinese Medicine
