# Supplementary material for: Analysis of Gut Microbiota as a Diagnostic Biomarker for Lung Adenocarcinoma with Qi-Deficiency and Phlegm-Turbid Stagnation
Source: Comb Chem High Throughput Screen. 2024 Jun 5;28(7):1240–50. doi: 10.2174/0113862073303081240521083505 (PMC12307956; doi:10.2174/0113862073303081240521083505)
Supplement: Supplementary file 1 [file CCHTS-28-7-1240_SD1.pdf]

Supplementary Material

Analysis of Gut Microbiota as a Diagnostic Biomarker for Lung Adenocarcinoma with Qi-Deficiency and Phlegm-Turbid Stagnation

Jiabin Chen[1,2,#], Qinqin Hu[1,#], Kequn Chai[1,*] and Sheng Wang[3,*]

[1]Department of Oncology, Tongde Hospital of Zhejiang Affiliated to Zhejiang University of Traditional Chinese Medicine, Hangzhou, Zhejiang 310012, China; [2]The Second Clinical Medical College, Zhejiang Chinese Medicine University, Hangzhou, Zhejiang 310012, China; [3]Respiratory Department, Jinhua Guangfu Cancer Hospital, Zhejiang 310053, China

STROBE Statement—checklist of items that should be included in reports of observational studies.

	Item No.	Recommendation	Page No.	Relevant text from manuscript
**Title and abstract**	1	(*a*) Indicate the study's design with a commonly used term in the title or the abstract	1	Analysis of gut microbiota as a diagnostic biomarker for Lung adenocarcinoma with Qi-deficiency and Phlegm-turbid stagnation
		(*b*) Provide in the abstract an informative and balanced summary of what was done and what was found	2	A diagnostic model was developed, which would be an available tool for clinical diagnosis of LUAD with QP syndrome.
Background/rationale	2	Explain the scientific background and rationale for the investigation being reported	3	Paragraphs 1 to 5
Objectives	3	State specific objectives, including any prespecified hypotheses	4	However, up to now, there has been no research indicating the composition and distribution of gut microbiota in LUAD patients with QP syndrome.
Study design	4	Present key elements of study design early in the paper	4	2.1 Subjects
Setting	5	Describe the setting, locations, and relevant dates, including periods of recruitment, exposure, follow-up, and data collection	None	
Participants	6	a) ***Cohort study***—Give the eligibility criteria, and the sources and methods of selection of participants. Describe methods of follow-up<br>b) ***Case-control study***—Give the eligibility criteria, and the sources and methods of case ascertainment and control selection. Give the ra-	a	

		tionale for the choice of cases and controls<br>c) ***Cross-sectional study***—Give the eligibility criteria, and the sources and methods of selection of participants		
		d) ***Cohort study***—For matched studies, give matching criteria and number of exposed and unexposed<br>*e)* ***Case-control study***—For matched studies, give matching criteria and the number of controls per case	d	
Variables	7	Clearly define all outcomes, exposures, predictors, potential confounders, and effect modifiers. Give diagnostic criteria, if applicable	4	Methods: 2.4--2.6
Data sources/ measurement	8*	For each variable of interest, give sources of data and details of methods of assessment (measurement). Describe comparability of assessment methods if there is more than one group	*4-5*	Methods: 2.4--2.6
Bias	9	Describe any efforts to address potential sources of bias	none	
Study size	10	Explain how the study size was arrived at	none	
Quantitative variables	11	Explain how quantitative variables were handled in the analyses. If applicable, describe which groupings were chosen and why	6	2.7 Statistical analysis
Statistical methods	12	(*a*) Describe all statistical methods, including those used to control for confounding	6	2.7 Statistical analysis
		(*b*) Describe any methods used to examine subgroups and interactions	none	
		(*c*) Explain how missing data were addressed	none	
		(*d*) *Cohort study*—If applicable, explain how loss to follow-up was addressed<br>*Case-control study*—If applicable, explain how matching of cases and controls was addressed<br>*Cross-sectional study*—If applicable, describe analytical methods taking account of sampling strategy	6	2.7 Statistical analysis

		(*e*) Describe any sensitivity analyses	none	
Participants	13*	(a) Report numbers of individuals at each stage of study—eg numbers potentially eligible, examined for eligibility, confirmed eligible, included in the study, completing follow-up, and analysed	6	
		(b) Give reasons for non-participation at each stage	none	
		(c) Consider use of a flow diagram	none	
Descriptive data	14*	(a) Give characteristics of study participants (eg demographic, clinical, social) and information on exposures and potential confounders	6	3.1 Clinical Characteristics
		(b) Indicate number of participants with missing data for each variable of interest	none	
		(c) *Cohort study*—Summarise follow-up time (eg, average and total amount)	none	
Outcome data	15*	*Cohort study*—Report numbers of outcome events or summary measures over time	*none*	
		Case-control study—Report numbers in each exposure category, or summary measures of exposure	*none*	
		Cross-sectional study—Report numbers of outcome events or summary measures	*none*	
Main results	16	(*a*) Give unadjusted estimates and, if applicable, confounder-adjusted estimates and their precision (eg, 95% confidence interval). Make clear which confounders were adjusted for and why they were included	*none*	
		(*b*) Report category boundaries when continuous variables were categorized	none	
		(*c*) If relevant, consider translating estimates of relative risk into absolute risk for a meaningful time period	*none*	
Other analyses	17	Report other analyses done—eg analyses of subgroups and interactions, and sensitivity analyses	*none*	

Key results	18	Summarise key results with reference to study objectives	10	Discussion section paragraphs 2 and 3
Limitations	19	Discuss limitations of the study, taking into account sources of potential bias or imprecision. Discuss both direction and magnitude of any potential bias	11	Discussion section paragraphs 4
Interpretation	20	Give a cautious overall interpretation of results considering objectives, limitations, multiplicity of analyses, results from similar studies, and other relevant evidence	11	Discussion section paragraphs 4
Generalisability	21	Discuss the generalisability (external validity) of the study results	none	
Funding	22	Give the source of funding and the role of the funders for the present study and, if applicable, for the original study on which the present article is based	13	

***Give information separately for cases and controls in case-control studies and, if applicable, for exposed and unexposed groups in cohort and cross-sectional studies.**

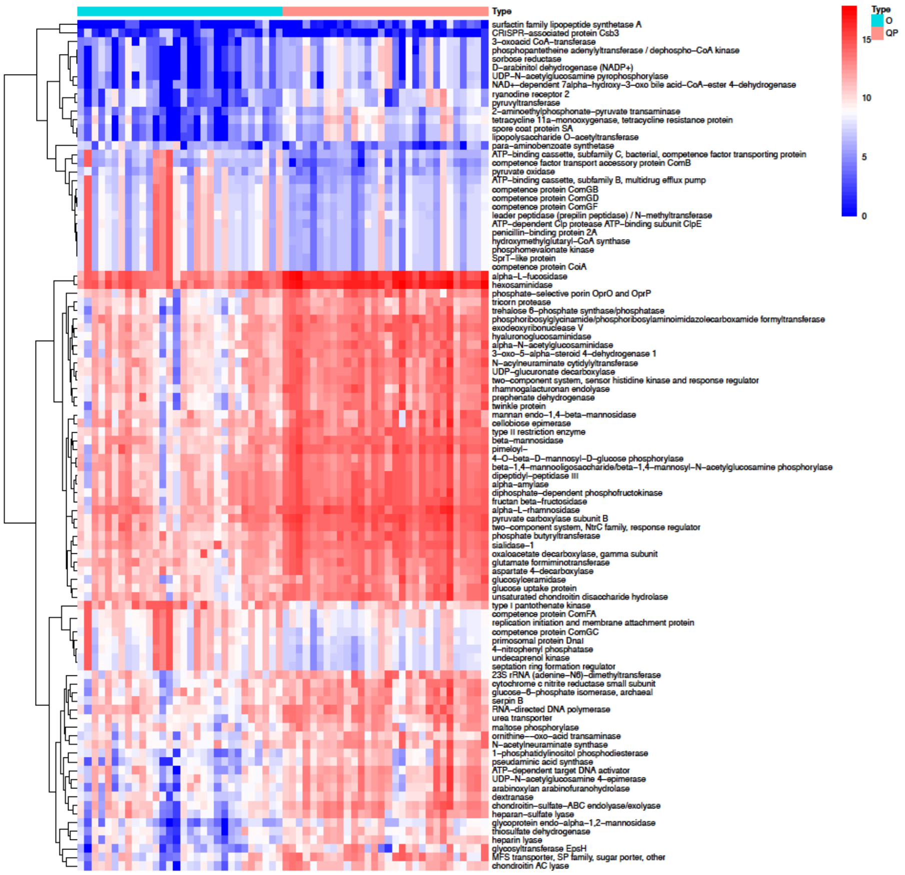

Fig. (S1).

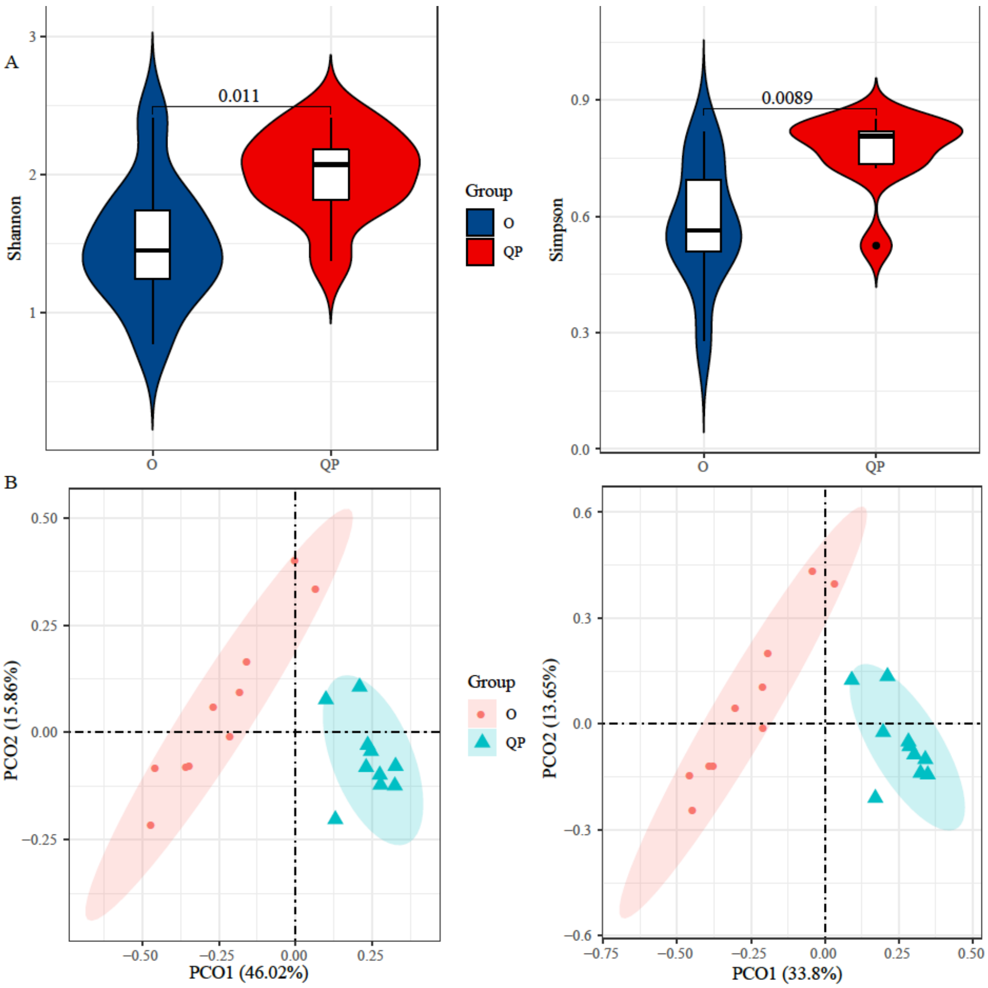

Fig. (S2).

Table 1. The basic information of all participants.

	16s-RNA			Metagenomics	
	**H**	**O**	**QP**	**O**	**QP**
Age	59.3±7.56	57.8±10.08	58.9±11.55	60.8±11.02	59.3±10.73
Sex(Female/Male)	18/12	12/18	9/21	6/4	5/5
Stage(I,II/III,IV)	-	21/9	16/14	4/6	6/4

H: healthy individuals; QP: LUAD with Qi-deficiency and Phlegm-turbid stagnation; O: LUAD with other syndrome.
